# Study of Concentrated Short Fiber Suspensions in Flows, Using Topological Data Analysis

**DOI:** 10.3390/e23091229

**Published:** 2021-09-18

**Authors:** Rabih Mezher, Jack Arayro, Nicolas Hascoet, Francisco Chinesta

**Affiliations:** 1 College of Engineering and Technology, American University of the Middle East, Egaila 54200, Kuwait; rabih.mezher@aum.edu.kw (R.M.); Jack.Arayro@aum.edu.kw (J.A.); 2 PIMM Lab, ESI Group Chair, Arts et Metiers Institute of Technology, 151 Boulevard de Hopital, 75013 Paris, France; Nicolas.Hascoet@ensam.eu

**Keywords:** topological data analysis (TDA), reinforced polymers, concentrated suspensions, flow induced orientation, discrete numerical simulation

## Abstract

The present study addresses the discrete simulation of the flow of concentrated suspensions encountered in the forming processes involving reinforced polymers, and more particularly the statistical characterization and description of the effects of the intense fiber interaction, occurring during the development of the flow induced orientation, on the fibers’ geometrical center trajectory. The number of interactions as well as the interaction intensity will depend on the fiber volume fraction and the applied shear, which should affect the stochastic trajectory. Topological data analysis (TDA) will be applied on the geometrical center trajectories of the simulated fiber to prove that a characteristic pattern can be extracted depending on the flow conditions (concentration and shear rate). This work proves that TDA allows capturing and extracting from the so-called persistence image, a pattern that characterizes the dependence of the fiber trajectory on the flow kinematics and the suspension concentration. Such a pattern could be used for classification and modeling purposes, in rheology or during processing monitoring.

## 1. Introduction

Reinforced polymers are widely used in industry for enhancing mechanical and functional performances while keeping the cost reasonable. The main issue related to the use of fiber-based reinforced polymers for elaborating short fiber composites is due to the difficulty of accurately predicting the flow induced orientation, with the final properties becoming strongly dependent on the final orientation state of fibers in the formed part.

The orientation evolution of an ellipsoidal fiber immersed in a flow characterized by a gradient of velocity can be computed by using the so-called Jeffery equation [1]. However, as soon as the fiber concentration increases, intense interactions between the rotating fibers takes place and the orientation kinematics of each fiber will differ from the one predicted by the Jeffery model.

At the population level (ensemble of fibers in a representative volume in which the velocity gradient is assumed almost identical) the interactions can be described as a diffusion term acting on the fiber orientation probability distribution Ψ, whose evolution is governed by the so-called Fokker-Planck equation [2], and more concretely the so-called Folgar-Tucker model [3]. Due to the fact that the orientation distribution depends on the physical coordinates (space and time) and also on the configurational ones (the orientation p defined on the surface of the unit sphere), Ψ(x,t,p), descriptions based on the moments of the orientation distribution are preferred [2,4]. Thus, the second order moment of the orientation distribution function reads:

(1) a(x,t)=∮p⊗pΨ(x,t,p)dp,

where ⊗ refers to the tensor product.

When considering a description based on the orientation tensors (orientation distribution moments), the diffusion term describing fiber interaction within the Folgar & Tucker formulation, results in a sort of randomizing term that tends, to evolve the orientation towards the isotropic state, that is a→I/3 (in 3D), with I the identity tensor [2,4].

However, many hypotheses were introduced when deriving the models describing the fiber interaction, fact that limits their validity and accuracy. Discrete simulations consider a population of fibers, subjected to two main actions, the hydrodynamic ones induced by the fluid flow, flow that is assumed unperturbed by the fibers presence and their orientation state, and the forces that apply when two neighbor fibers approach mutually activating, first hydrodynamics forces and then contact forces for avoiding interpenetration.

Discrete simulations are extremely expensive because of the high number of fibers to be considered for representing the different concentration regimes, and because of the extremely small time steps that the small length scales involved by the fibers interaction imply.

When fibers enter in contact, having a non-null relative velocity, the interaction will affect the orientation kinematics from one side, but it will also affect the fibers geometrical center trajectory. Thus, it is postulated that this trajectory will depend on the number and intensity of the fiber interaction, both expected scaling with the flow gradient of velocity, the fiber concentration and the orientation state.

Thus, the analysis of those erratic trajectories that the fiber follow, should provide a very valuable information on the orientation state (difficult to measure in 3D flows of concentrated suspensions), the local concentration that could differ from one point to another in the flow, or even the effective velocity gradient that could differ from the nominal one, that as previously indicated is assumed the one unperturbed by the fibers presence.

However, extracting information from those erratic trajectories seems difficult, needing the use of adequate metrics to compare them, that apparently seem very different even when the flow conditions remain identical. Moreover, the usual statistical descriptors (widely considered for describing roughness for instance) seem insufficient for describing the trajectory richness. Thus, robust metrics for describing in a concise, compact and rich enough way, with the suitable invariance properties, are needed for making possible unsupervised clustering and supervised classification of the different trajectories. For that purpose, the present work considers topological data analysis for analyzing the stochastic time-series induced by the fiber interactions, at the level of the movement of the fiber geometrical center.

The paper is organized as follows. Section 2 describes the discrete simulation of flows involving concentrated fiber suspensions. Then, in Section 3, the so-called Topological Data Analysis (TDA) will be revisited. Finally, the numerical results will be reported in Section 4, before addressing some final concluding remarks in Section 5.

## 2. Discrete Simulation

The main assumptions considered in the the modelling and simulation framework are [5,6]:

1. The suspending fluid is Newtonian, incompressible and the flow is laminar;
2. The fluid velocity gradient is assumed being homogeneous in the considered representative volume where the calculations are performed, with the velocity field assumed unperturbed by the particles presence and their orientation;
3. The mass of the fibers is negligible, thus the inertia of the fibers is neglected;
4. Fibers are considered to have the same length, but they could have different length;
5. The long-range hydrodynamic interactions are considered along with short-range hydrodynamic interactions between fibers;
6. Initially and before the simulation starts, the fibers are homogeneously and almost isotropically distributed in the considered volume, with interpenetration prevented.

The position of the geometrical center *G* of fiber (α), $r^{\left(\alpha \right)}$, is given by

(2) $$r^{\left(\alpha \right)}=x^{\left(\alpha \right)}x+y^{\left(\alpha \right)}y+z^{\left(\alpha \right)}z,$$

where x, y and z represent respectively the three unit vectors related to the three space coordinates.

Fibers are assumed having an ellipsoidal shape, with length *l* and diameter *d* (taken at the axis center). Thus, the aspect ratio of the fibers *r* reads:

(3) $$r=\frac{l}{d}.$$

In the numerical simulations described later, the considered fibers have an aspect ratio of 20. Thus, the fibers will be represented by elongated ellipsoids, whose orientation will be described by a unit vector p aligned with the ellipsoid longest axis. Moreover, the considered aspect ratio allows assuming the fibers rigid, as experimental observations prove for usual materials, as for example glass fibers.

Since the suspensions are considered concentrated, with the fiber volume fraction noted by ϕ, the following inequality applies:

(4) $$\phi \geq \frac{1}{r}.$$

The higher *r* (i.e., long fibers), the more the system is considered concentrated for a fixed fibers concentration ϕ. In what follows, the fibers are supposed to be sufficiently long (i.e., r≫1), approaching the cylindrical shape.

The fixed frame is defined from $\left(O,x,y,z\right)$, whereas another frame is attached to each fiber: $\left(G,x^{\prime },y^{\prime },z^{\prime }\right)$. A shear flow is applied, with the velocity field expressed from

(5) $$V^{T}(x)=\left(V_{1},V_{2},V_{3}\right)=\left(\dot{\gamma }y,0,0\right),$$

with $\dot{\gamma }$ the applied shear rate and *y* the *y*-coordinate of the fiber geometrical center. This expression allows defining the velocity gradient ∇V as well as its symmetric and skew-symmetric parts, D and W respectively, with $\Omega =\frac{1}{2}\left(\nabla \times V\right)$.

The fiber orientation is defined by the unit vector $p^{\left(\alpha \right)}$ such as $p^{\left(\alpha \right)}=p_{1}^{\left(\alpha \right)}x+p_{2}^{\left(\alpha \right)}y+p_{3}^{\left(\alpha \right)}z$. The relative fluid/particle velocity at *G* reads

(6) $${\dot{q}}^{\left(\alpha \right)}={\dot{r}}^{\left(\alpha \right)}-V\left(r^{\left(\alpha \right)}\right)={\dot{r}}^{\left(\alpha \right)}-\dot{\gamma }y^{\left(\alpha \right)}x.$$

### 2.1. Fiber Motion Equations: Translation

The net force that the fluid transfer to the fiber scales with the relative velocity at *G* from the so-called resistance tensor ζ, and then the force balance with the acting force F, reads

(7) $$F^{\left(\alpha \right)}+\zeta^{\left(\alpha \right)}\cdot {\dot{q}}^{\left(\alpha \right)}=0,$$

where the friction tensor expression is given in [7], and depends on the fluid viscosity, the fiber geometry and its orientation.

### 2.2. Fiber Motion Equations: Rotation

First we consider the dilute case where fiber interaction cans be neglected. The fluid deformation induces on the fiber the torque $H^{\left(\alpha \right)}:D$ (with H a third order resistance tensor) and the fluid/fiber relative rotary velocity $\omega^{\left(\alpha \right)}$ induces the torque $\xi^{\left(\alpha \right)}\cdot \omega^{\left(\alpha \right)}$, with $\xi^{\left(\alpha \right)}$ a second order resistance tensor. Both resistance tensors [7] depend again on the fluid viscosity, fiber geometry and fiber orientation.

When neglecting inertia effects, the torque balance (in absence of fiber interactions) reads

(8) $$\xi^{\left(\alpha \right)}\cdot \omega^{\left(\alpha \right)}+H^{\left(\alpha \right)}:D=0,$$

from which the fiber rotary velocity can be extracted,

(9) $${\dot{p}}^{\left(\alpha \right)}=-p^{\left(\alpha \right)}\times \left(\omega^{\left(\alpha \right)}-\Omega \right),$$

that for infinite aspect ratio fibers leads to

(10) $${\dot{p}}^{\left(\alpha \right)}={\dot{p}}_{J}^{\left(\alpha \right)}=W\cdot p^{\left(\alpha \right)}+\left[D\cdot p^{\left(\alpha \right)}-\left(D:p^{\left(\alpha \right)}⊗p^{\left(\alpha \right)}\right)p^{\left(\alpha \right)}\right],$$

that coincided with the Jeffery equation [1].

When the suspension becomes concentrated enough, fiber-fiber interactions occur. Thus, short-range forces will appear on the fibers as they interact.

There are two types of interactions considered via two types of forces: A lubrication force $F_{lb}$ occurs when two fibers approach one another; and a contact force $F_{c}$ when they touch, that when neglecting friction (the roughness of the fiber surface is very small, fact that enables neglecting the induced friction force), the contact force, as well as the lubrication one, is assumed acting in the normal direction.

The resulting interaction force on fiber (α) reads:

(11) $$F^{\left(\alpha \right)}=\sum_{\beta \neq \alpha }F_{c}^{\left(\alpha ,\beta \right)}n^{\left(\alpha ,\beta \right)}+\sum_{\mu \neq \alpha }F_{lb}^{\left(\alpha ,\mu \right)}n^{\left(\alpha ,\mu \right)},$$

that will induce a torque $T^{\left(\alpha \right)}$ on the considered fiber, leading to the torque balance

(12) $$T^{\left(\alpha \right)}+\xi^{\left(\alpha \right)}\cdot \omega^{\left(\alpha \right)}+H^{\left(\alpha \right)}:D=0,$$

from which

(13) $$\omega^{\left(\alpha \right)}=-\xi^{{\left(\alpha \right)}^{-1}}\cdot \left(T^{\left(\alpha \right)}+H^{\left(\alpha \right)}:D\right),$$

leading to the fiber rotary velocity ${\dot{p}}^{\left(\alpha \right)}$.

Thus, knowing the resulting force applied on fiber (α) one can compute the relative velocity at *G*, ${\dot{q}}^{\left(\alpha \right)}$ (that allows updating the fiber center position), and the fiber rotary velocity ${\dot{p}}^{\left(\alpha \right)}$.

The calculation of the distance between two fibers and the calculation of the lubrication forces depending on the approaching velocity ${\dot{\Theta }}^{\left(\alpha ,\beta \right)}$, were detailed in [8].

Contact forces are assumed to occur if the gap between two close fibers is equal to zero and if $F_{c}^{\left(\alpha ,\beta \right)}\neq 0$. The condition employed in the present work reads [9]

(14) $$\frac{d}{dt}\left[\left(r^{\left(\alpha \right)}-r^{\left(\beta \right)}\right)\cdot n^{\left(\alpha ,\beta \right)}\right]={\dot{\Theta }}^{\left(\alpha ,\beta \right)}=0,$$

with $\Theta^{\left(\alpha ,\beta \right)}\approx 0$. It physically means that two fibers in contact cannot penetrate one another.

For solving the problem, fibers are grouped. Imagine that fibers (α) and (β) are in contact. The first group is composed by all the fibers in interaction with fiber (α). The second group is all the fibers in interaction with fiber (β). There is one unknown force for each pair of fibers, because the forces acting on the two fibers are equal in magnitude but opposite in direction. All forces for these two groups are coupled and should be solved together with all the interactions in the suspension by enforcing the kinematic constraints (14) at each contact level. For additional details the interested reader can refer to [5] and the references therein.

## 3. Topological Data Analysis

Data is generated by considering a population of fibers inside a computational box, that represents the so-called representative volume. The number of fibers depends on the considered fibers volume fraction (fibers concentration). Then, a simple shear flow is assumed taking place inside, with the velocity given by $V^{T}=\left(\dot{\gamma }y,0,0\right)$. As discussed above, the flow is assumed unperturbed by the fibers presence and orientation. In absence of interactions, the geometrical center of each fiber will follow a rectilinear trajectory, traversing the computational box, until leaving it from its right boundary. Instead of increasing the box size, fully periodic boundary conditions are enforced. Thus, as soon as a fiber leaves the box from its right boundary it is re-injected into the box through its left boundary. The computational cell perfectly represents the bulk flow conditions, as soon as the analyzed flow is not affected by the physical walls (e.g., the mould walls). Here, we assume that the flow cell (representative volume) is far enough (with respect to the fiber length) from the physical walls for ignoring the effects of those walls.

In the absence of interactions, the orientation of each fiber describes the so-called Jeffery orbit. When concentration increases, Jeffery orbits intersect one another and then lubrication and contact forces appear when fibers interact. The number of interactions will scale with the fiber concentration, while the interaction intensity scales with the applied shear rate. Thus, the higher the fibers’ concentration and the applied shear rate, the more intense and frequent the interactions occurring in the flow, creating a strong perturbation in the orientation kinematics (fiber rotary velocity) as well as in the erratic trajectory described by the fiber centers.

The interactions (lubrication and contacts) occur inside the box, but due to the assumed and enforced periodic boundary conditions, fibers located in the neighborhood of the right boundary can interact with the ones located in the neighborhood of the left one, and those close to the bottom boundary with the ones close to the top one, and similarly for the front and rear sides of the box.

Fibers are initially located randomly into the box, while avoiding interpenetration. Thus, at the end of the box filling an almost isotropic orientation state is obtained, i.e., a≈I/3.

A test fiber is considered close to the center of the box, and its trajectory is recorded, in particular the three components of the fluctuating vector $\dot{q}$ acting on it, that will represent the three time series $S_{x},S_{y},S_{z}$: $S_{x}=\left\{{\dot{q}}_{x}^{1},{\dot{q}}_{x}^{2},\ldots \right\}$ and similarly for the other two times series. In these time series and for comparison purposes, the exponent refers to the quantity of applied strain, i.e., ${•}^{n}$ refers to $•(\dot{\gamma }t_{n})$.

The kinematics of the test particle is followed a certain time, in order to almost cover the three main regimes that it is experiencing:

1. The first regime is the one taking place at the very beginning when the flow starts, where the initial fiber distribution evolves in absence of interactions, until fibers approaching ones another induce the expected fiber interaction (lubrication and contact);
2. The second regime is the one when the orientation of the fibers in the population evolve, trying to align with the flow direction (induced by the applied shear) but in presence of numerous and intense interactions;
3. The third region is an almost stablished regime, when fibers are quite aligned with the preferential orientation direction (the x-coordinate in the case here studied). In this case the number of interaction reduces because when fibers are almost aligned in the same direction, interactions are much less probable and much less intense. However, as the fibers are ellipsoids, they cannot align in a stable manner in the flow direction, the rotary velocity never vanishes, even in absence of interactions. The rotary velocity becomes very small when ellipsoids align along the flow direction, and consequently the fiber spend a lot of time aligned in the flow direction, but it continues its rotation, and the rotary velocity increases when the orientation moves apart form the flow direction, reaching its maximum velocity when the *y*-coordinate reaches its maximum value. Then, the rotary velocity decreases again when the fiber orientation approaches again the flow direction, and the cycle repeats and rotation continues. Thus, the fiber spend long periods almost aligned with the flow, and rotate very fast outside this most stable direction (the local orientation with the flow). During this fast rotation the interactions are numerous and intense, because of the fact that each fiber rotates at different instants.

In order to compare the just referred time series, we must consider appropriate metrics able to find the similarity of times series, neither identical nor superposable. Topological Data Analysis [10,11,12] inherits the invariance properties of topology, and then it is an appealing candidate for analyzing, describing and finally classifying time series with respect to the concentration regime and the applied shear rate.

For the sake of clarity, we will consider a generic time series $S=\{s^{1},s^{2},\ldots ,s^{k}\ldots \}$. To extract the topology of the data composing the time series, first the extremum points (local minimums and local maximums) are identified, and then we proceed to the one-to-one local-minimum/local-maxixum neighbors pairing. In the pairing process, when multiple alternatives exist, the one maximizing the max to min distance is retained.

Now, we assume that P min-max pairs have been constructed: $\left(b^{1},d^{1}\right),\ldots ,\left(b^{P},d^{P}\right)$, where *b* refers to the minimum, also referred as *birth*, and *d* refers to the maximum, or the *death*. Each one of these pairs results in a point in the so-called persistence diagram, with the birth component reported in its horizontal axis and the deaths in the vertical one. Being the maximum always greater (or equal) than the minimum, points will group in the upper part with respect to the bisector (diagonal of the square birth/death representation).

Instead of representing on the vertical axis the deaths, an alternantive derived representation consists of representing the lifetime, that is, $d^{k}-b^{k}$. Thus, the points reported into the so-called life-time diagram are the P data points: $\left(b^{1},d^{1}-b^{1}\right),\ldots ,\left(b^{P},d^{P}-b^{P}\right)$, that now appear distributed everywhere in the 2D representation.

Calculating distances between clouds of points is possible when using an adequate metrics. One possibility consists of using the Wasserstein metrics usually employed in optimal transport [13], that first matches the points of all the considered sets, in order to minimize the cost related to the distance among them, and then compute the Euclidian distance between the matched points-sets.

However, using this kind of data representation in usual artificial intelligence and machine learning techniques for clustering, classifying and modeling (i.e., constructing regressions) remains its trickiest issue. For that reason, a step forward consists of transforming the life-time diagram into the so-called persistence image, defined in a vector space facilitating its post-processing for a diversity of purposes.

For that purpose, and as described in our former works [14,15,16], we associate to each data-point in the life-time diagram a bivariate normal distribution, weighted and then integrated in different patches on a square domain covering the support of the regularized life-time diagram, leading to the so-called persistence images.

The resulting persistence images have an important property, the one of be invariant for time-series having similar topologies, even when they cannot be perfectly matched when using their time-representations.

Thus, persistence images enable efficient unsupervised clustering or supervised classification, and can be used also as input in regressions, by considering convolutional neural networks –cNN– directly applied on them, or nonlinear polynomial regression applied on the coefficients of their PCA decomposition [17].

## 4. Results

According with the rationale described at the beginning of Section 3, different time series related to the movement of a test fiber in different flow conditions, the last characterized by the fibers volume fraction (%) and the applied shear rate s^−1^, were generated. The considered design of experiments –DoE– is given in Table 1.

The initial orientation is almost isotropic, that is, there are fibers pointing in any direction of the unit sphere, with an almost a uniform distribution. Thus, one expects that when the flow starts, the flow induced orientation, trying to align all the fibers along the flow direction (as discussed before), will create frequent and intense fiber-fiber interactions, scaling with the shear rate and the concentration. These interactions will induce significant displacements of the fibers geometrical centers. When the fibers align along the flow direction, i.e., with the velocity field, they remain most of the time aligned with the flow. However the alignment with the flow is never permanent because of two main reasons.

First, when considering fibers modeled by ellipsoids (as it is the case here) the local alignment is not a steady solution (no steady solution exists). The rotary velocity reaches its smallest value when the fiber is aligned with the flow, but it is not exactly zero. Thus, the fiber moves apart from the alignment with the flow, to make a turn, coming back to the alignment with the flow, where again it spends a long period before starting another rotation, and so on.

The second advocated reason is, that even if the interaction is much less intense when fibers are globally quite aligned with the flow, the sporadic rotations just described create fiber-fiber interactions that induce the displacement of the fibers geometrical centers, while the orientation also deviate from the local alignment with the flow.

Moreover, as fibers are rotating according to the applied shear, in the clockwise direction in our case, the displacement of the fibers geometrical center is expected exhibiting an asymmetric behavior.

**Figure 1:**
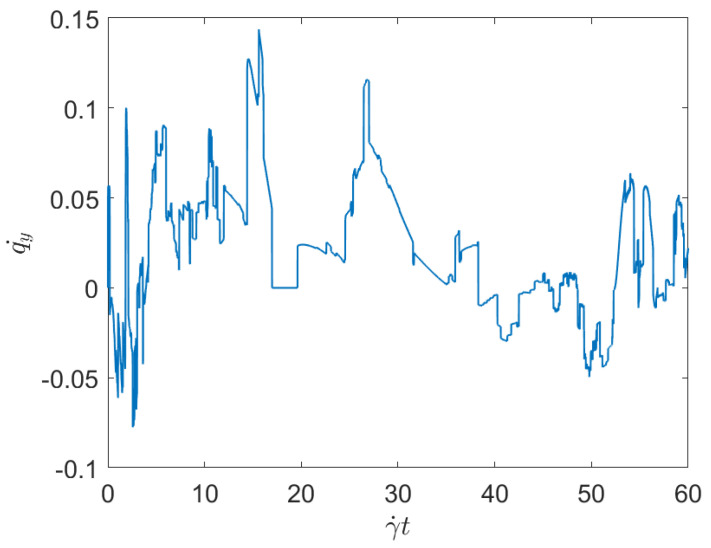
Time series related to displacement on the *y*-direction in Case 4: Minimum shear rate.

**Figure 2:**
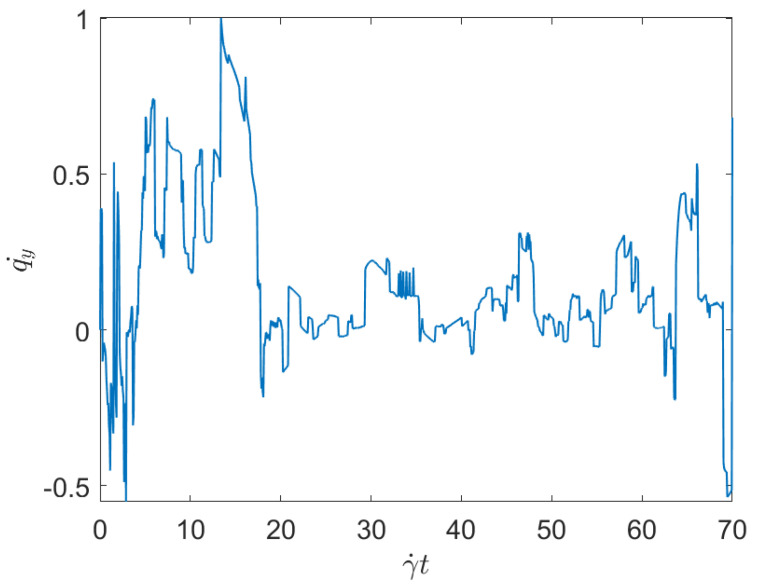
Time series related to displacement on the *y*-direction in Case 6: Maximum shear rate.

For confirming the previous expectations, we consider Cases 4 and 6 in Table 1, related to the minimum and maximum applied shear rates, both having the same fiber concentration, and compares the displacement of the test fiber geometrical center along the *y*-direction (the shear direction), both cases represented respectively in Figure 1 and Figure 2. These two figures prove that the larger is the shear rate, the higher is the fiber-fiber interaction intensity, and consequently the displacement induced on the fibers along the shear direction (*y*-direction—with the flow occurring along the *x*-direction).

**Figure 3:**
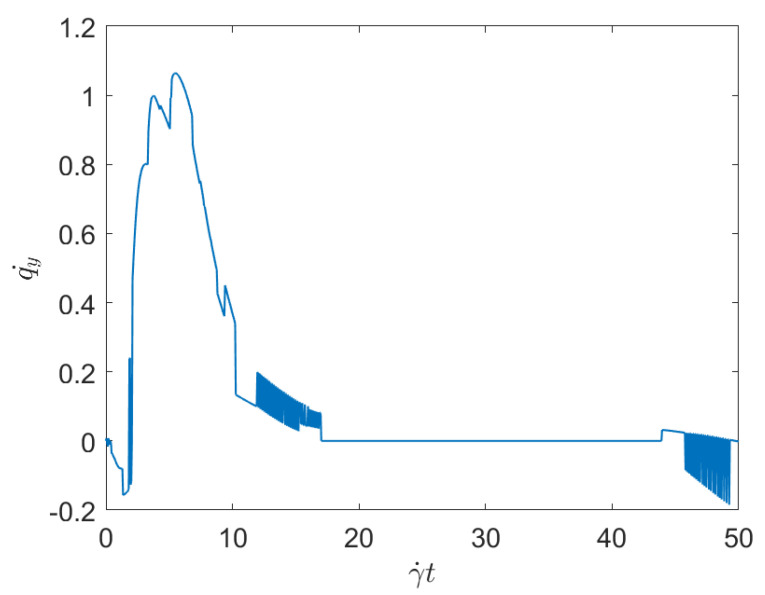
Time series related to displacement on the *y*-direction in Case 1: Minimum concentration.

**Figure 4:**
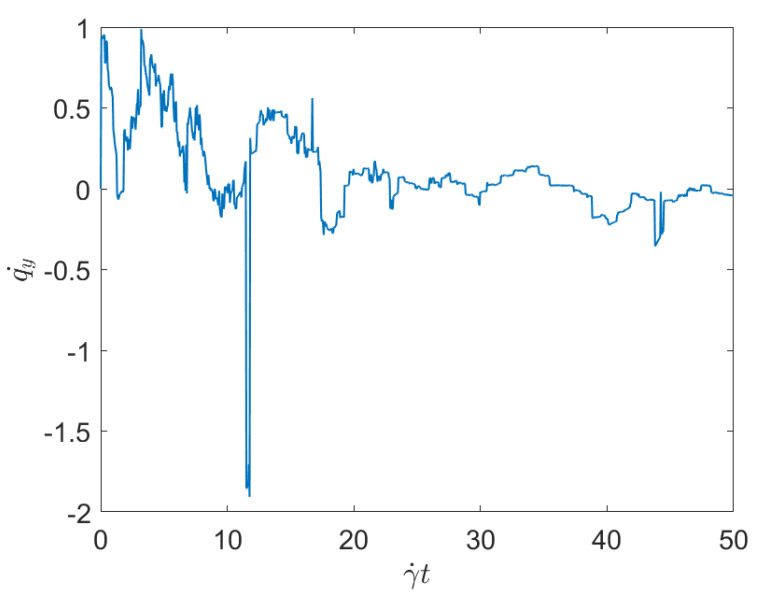
Time series related to displacement on the *y*-direction in Case 3: Maximum concentration.

To evaluate the effect of the concentration while keeping constant the applied shear, we consider Case 1 and Case 3, with respectively the minimum and maximum fibers concentration (both subjected to the same applied shear rate). Figure 3 and Figure 4 represent the associated displacement along the shear direction (*y*-coordinate). As it can be noticed from the observation of these figures, for the lower concentration, after the numerous interactions that follow the flow initiation, a plateau corresponding to the fibers alignment along the flow direction, where interactions almost disappear, is noticed. As discussed previously, fibers move apart form the local alignment for performing a full rotation before coming back again to the orientation with the flow, in which it stays for a long period (the rotary velocity is minimum when fibers are almost aligned with the flow). For the maximum concentration, fiber-fiber interactions persist after the transient regime, and the permanent regime continues exhibiting intense fluctuations induced by the interactions. It can be stressed that the concentration mainly affects the number of interactions, but their intensity seems more influenced by the shear rate than by the fiber concentration.

**Figure 5:**
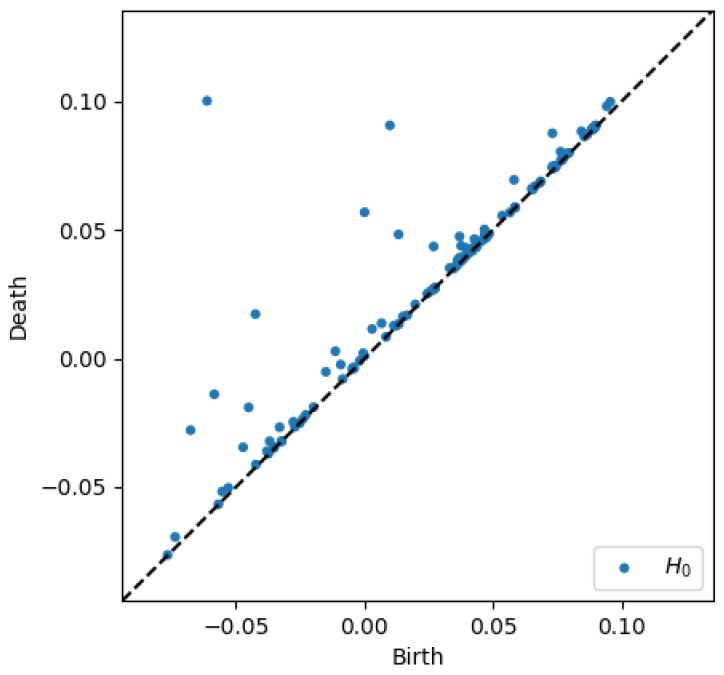
Persistence diagram related to displacement on the *y*-direction in Case 4: Minimum shear rate.

**Figure 6:**
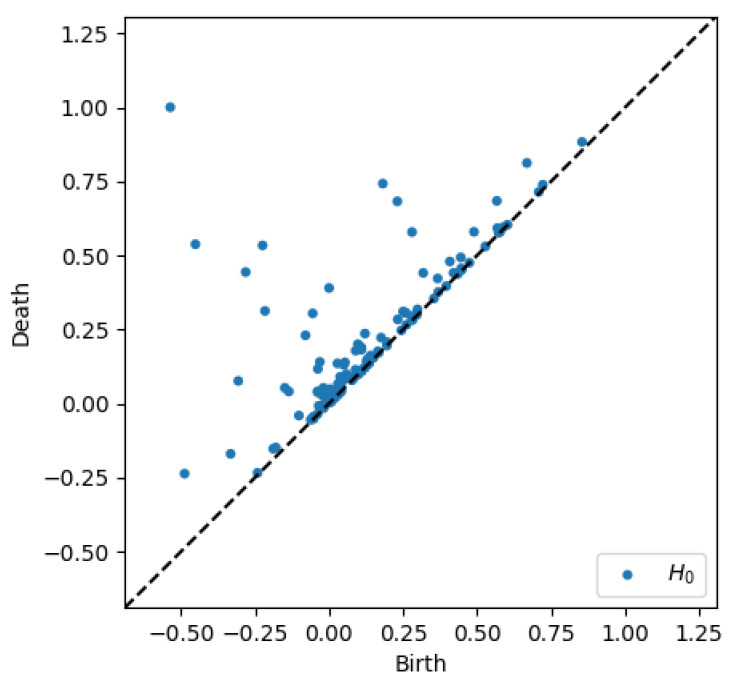
Persistence diagram related to displacement on the *y*-direction in Case 6: Maximum shear rate.

**Figure 7:**
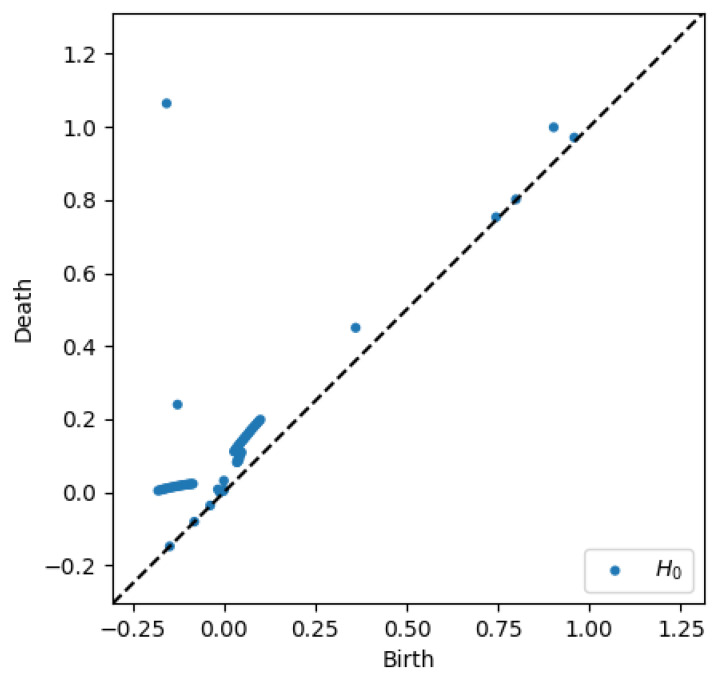
Persistence diagram related to displacement on the *y*-direction in Case 1: Minimum concentration.

**Figure 8:**
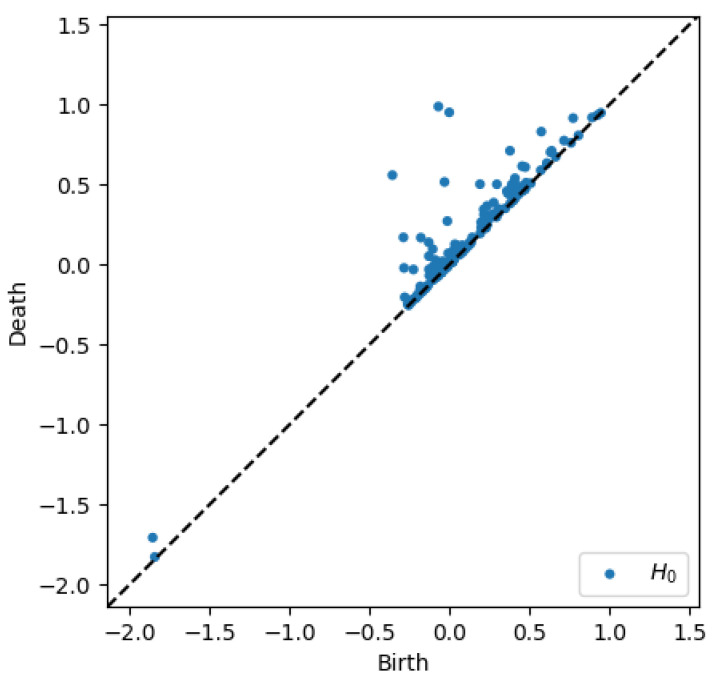
Persistence diagram related to displacement on the *y*-direction in Case 3: Maximum concentration.

To better appreciate the number and distribution of the topological events, Figure 5, Figure 6, Figure 7 and Figure 8 show the persistence diagrams related respectively to Figure 1, Figure 2, Figure 3 and Figure 4, where each blue dot represents a topological event, with its appearance reported in the *x*-coordinate axis and its death in its *y*-coordinate axis, being the vertical distance to the diagonal a representation of its persistence (its lifetime).

Figure 5 and Figure 6 clearly reveal that the topology becomes more persistent when increasing the shear rate, with the associated topological event appearance asymmetrically distributed with respect to the zero value. High shear rates induce strong interactions (as observed in Figure 2) that result in highly fluctuating dynamics, with large amplitudes, that result in persistent topology. On the contrary, when the shear rate decreases the fluctuations are much less intense (smaller amplitudes) inducing an ephemeral topology, with the topological events closer to the diagram diagonal.

Now, focusing on the effect of concentration, from Figure 7 and Figure 8 it can be stressed that in the dilute regime, represented by Figure 3, the largest persistent topology is associated with the transient regime, with ephemeral events occurring as soon as fibers almost align with the flow.

When the concentration increases nothing changes significantly, as expected, concerning the most persistent topology, however, the ephemeral one becomes more abundant and erratic than the one related to the dilute case. It is important to notice that the scale of representation is impacted by an isolated negative displacement that induces a displacement along the *x*-axis, and that could be considered as an outlier. These findings confirm that the concentration affects more the ephemeral events that the persistent topology.

Thus, two main scales can be differentiate, the one related to the transient regime, involving more persistent topology, and the one related to long-time regime exhibiting more ephemeral events.

The main issue, previously discussed, is the way of using a compact, concise and complete descriptor of the time series depicted in the previous figures (Figure 1, Figure 2, Figure 3 and Figure 4), more easy to manipulate than the discrete persistence diagrams reported in Figure 5, Figure 6, Figure 7 and Figure 8.

The use of persistence images is a valuable route for accomplishing it, because they allow extracting and differentiating micro and macro events, inducing ephemeral or persistent topology. Persistence images are defined in a vector space and can be easily manipulated by most of the state-of-the-art artificial intelligence and machine learning techniques. These images contain a rich multi-scale information able to represent the amount of topology and its persistence, expected describing the fibers trajectories depending on the concentration and shear rate, the former induing the amount of topological events and the last their persistence.

Figure 9 schematizes the persistence image content, where the horizontal axis refers to the value at which the topological event appears, while the vertical one refers to its persistence. Thus, Figure 10, Figure 11, Figure 12 and Figure 13 represent the persistence images associated respectively to Figure 1, Figure 2, Figure 3 and Figure 4, that describe the findings just discussed when referring to the associated persistence diagrams (Figure 5, Figure 6, Figure 7 and Figure 8).

**Figure 14:**
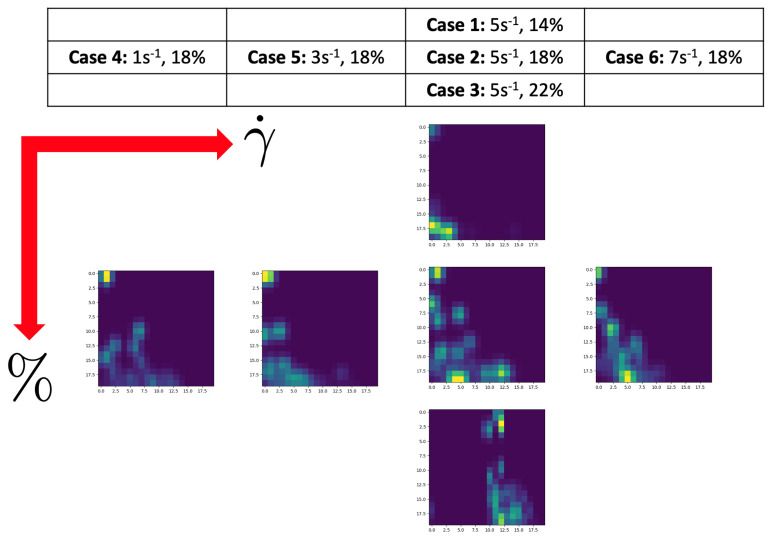
${\dot{q}}_{y}$ persistence images in different fibers volume fraction/shear rate conditions.

To sum up the effect of the concentration and the applied shear rate on the persistence image, Figure 14 represents the images corresponding to ${\dot{q}}_{y}$ in the different concentration/shear rate conditions, where a clear evolution of the topological pattern can be appreciated.

## 5. Conclusions

This paper proved that interactions affect, in a very precise way, the trajectory followed by the geometrical center of the interacting particles. Because of the high variability, a robust metric was chosen for comparison purposes, concretely topological data analysis. Thus, the time-series related to the erratic perturbation of the nominal trajectories, reflecting the interactions (lubrication and contact) allows to extract a sort of topological pattern, the so-called persistence image, that characterizes in a stable manner (invariant description) all the trajectories related to the same flow conditions, in particular same values of the fiber concentration and flow shear rate, both effecting the number and intensity of the interactions, and then having a noticeable effect on the trajectory topology.

This work opens numerous perspectives, in particular the one related to the flow monitoring, to infer, from the recorded trajectory, local quantities, like the the effective shear rate, concentration and ensemble orientation (moments of the orientation distribution function).

## Figures and Tables

**Figure 9:**
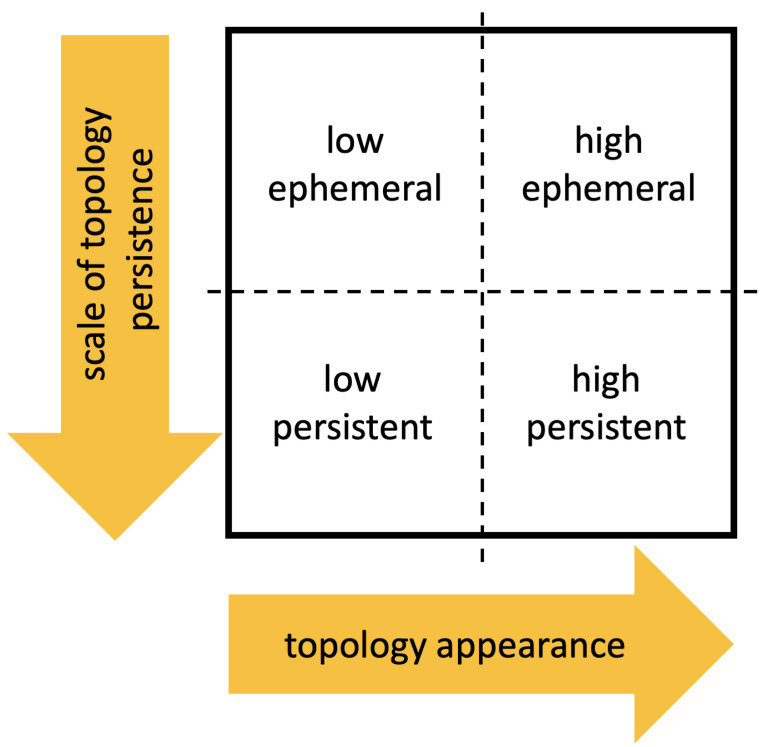
Persistence images reader code.

**Figure 10:**
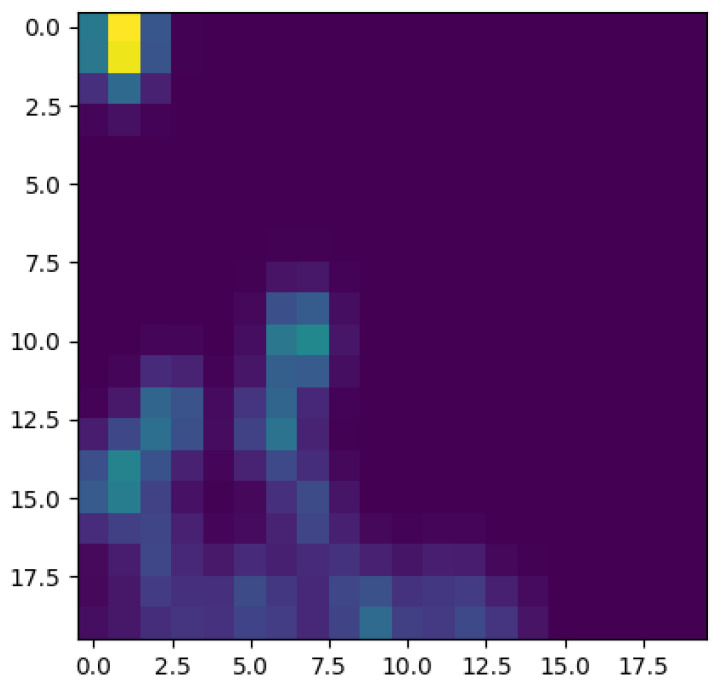
Persistence image related to displacement on the *y*-direction in Case 4: Minimum shear rate.

**Figure 11:**
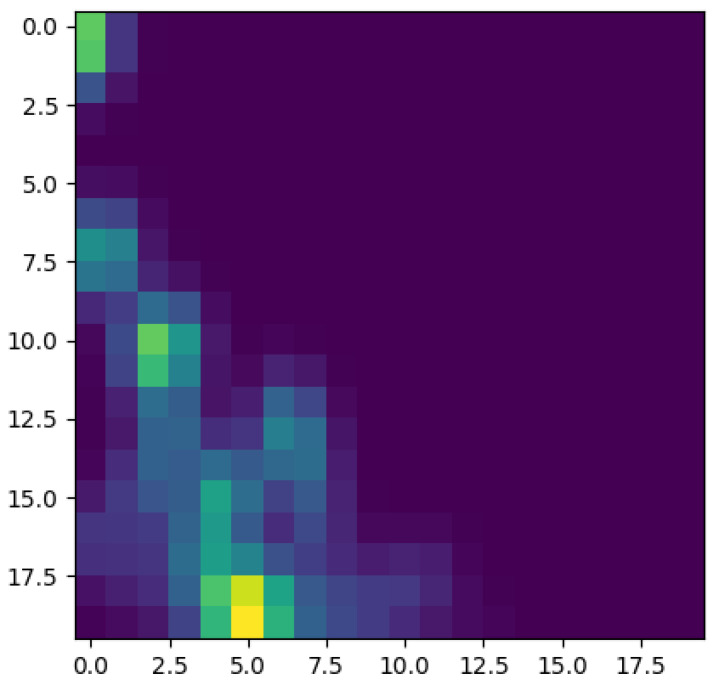
Persistence image related to displacement on the *y*-direction in Case 6: Maximum shear rate.

**Figure 12:**
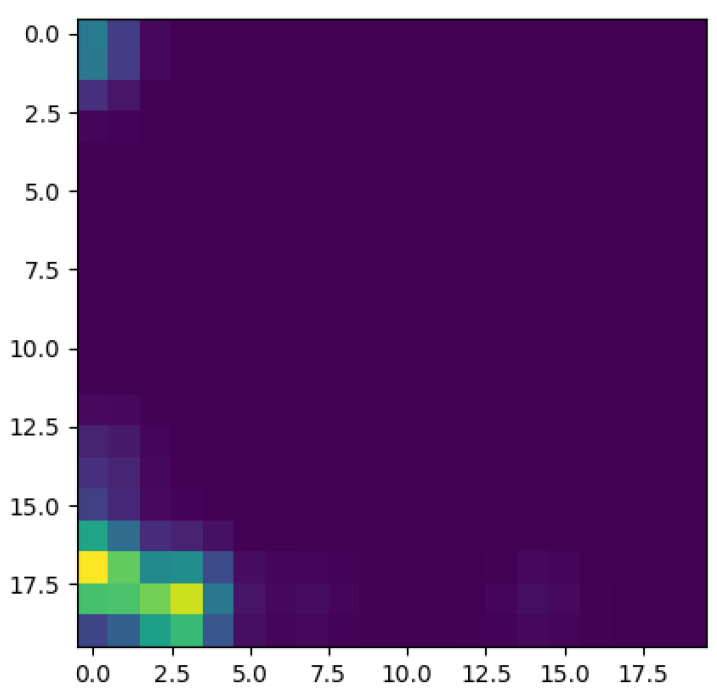
Persistence image related to displacement on the *y*-direction in Case 1: Minimum concentration.

**Figure 13:**
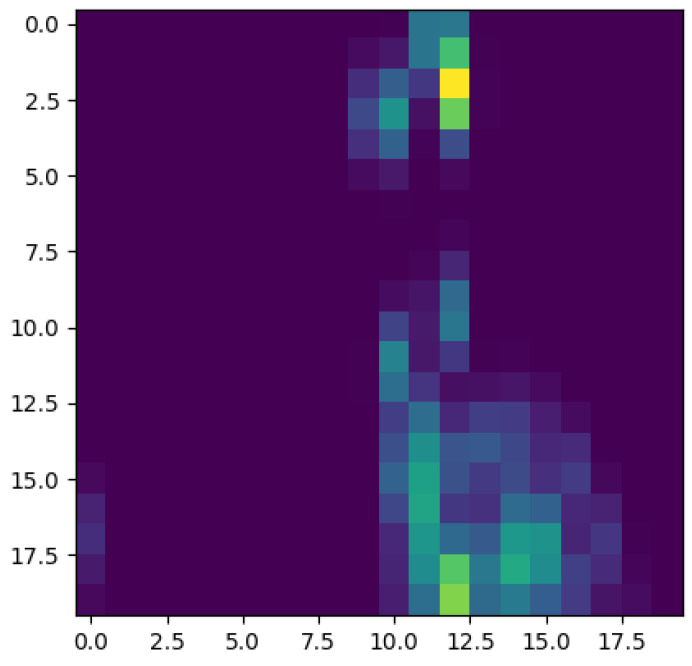
Persistence image related to displacement on the *y*-direction in Case 3: Maximum concentration.

**Table 1:** Design of Experiments.

Case	Concentration (%)	Shear Rate (s^−1^)
1	14	5
2	18	5
3	22	5
4	18	1
5	18	3
6	18	7

## Data Availability

Data is available under request.

## References

[1] Jeffery G.B. (1922). The motion of ellipsoidal particles immersed in a viscous fluid. Proc. R. Soc. Lond..

[2] Binetruy C., Chinesta F., Keunings R. (2015). Flows in Polymers, Reinforced Polymers and Composites: A Multiscale Approach.

[3] Folgar F., Tucker C. (1984). Orientation behavior of fibres in concentrated suspensions. J. Reinf. Plast. Comp..

[4] Advani S., Tucker C. (1987). The use of tensors to describe and predict fibre orientation in short fibre composites. J. Rheol..

[5] Mezher R., Abisset-Chavanne E., Ferec J., Ausias G., Chinesta F. (2015). Direct simulation of concentrated fiber suspensions subjected to bending effects. Model. Simul. Mater. Sci. Eng..

[6] Mezher R., Perez M., Scheuer A., Abisset-Chavanne E., Chinesta F., Keunings R. (2016). Analysis of the Folgar & Tucker model for concentrated fibre suspensions via direct numerical simulation. Compos. Part A.

[7] Kim S., Karrila S.J. (1991). Microdynamics: Principles and Selected Applications.

[8] Yamane Y., Kaneda Y., Doi M. (1994). Numerical-simulation of semidilute suspensions of rodlike particles in shear-flow. J. Non Newton. Fluid Mech..

[9] Ausias G., Fan X.J., Tanner R. (2006). Direct simulation for concentrated fibre suspensions in transient and steady state shear flows. J. Non-Newton. Fluid Mech..

[10] Rabadan R., Blumberg A.J. (2020). Topological Data Analysis For Genomics and Evolution.

[11] Oudot S.Y. (2010). Persistence Theory: From Quiver Representation to Data Analysis.

[12] Chazal F., Michel B. (2017). An introduction to Topological Data Analysis: Fundamental and practical aspects for data scientists. J. Société Française Stat..

[13] Peyré G., Cuturi M. (2019). Computational Optimal Transport. Found. Trends Mach. Learn..

[14] Frahi T., Chinesta F., Falco A., Badias A., Cueto E., Choi H.Y., Han M., Duval J.L. (2021). Empowering Advanced Driver-Assistance Systems from Topological Data Analysis. Mathematics.

[15] Frahi T., Yun M., Argerich C., Falco A., Chinesta F. (2020). Tape Surfaces Characterization with Persistence Images. AIMS Mater. Sci..

[16] Frahi T., Falco A., Vinh Mau B., Duval J.L., Chinesta F. (2021). Empowering Advanced Parametric Modes Clustering from Topological Data Analysis. Appl. Sci..

[17] Yun M., Argerich C., Cueto E., Duval J.L., Chinesta F. (2020). Nonlinear regression operating on microstructures described from Topological Data Analysis for the real-time prediction of effective properties. Materials.

